# Initial Validation of the Hungarian Version of Abridged Nutrition for Sport Knowledge Questionnaire (ANSKQ-HU)

**DOI:** 10.3390/sports13120422

**Published:** 2025-12-02

**Authors:** Réka Erika Kovács, Gusztáv József Tornóczky, Gina Louise Trakman, Szilvia Boros, István Karsai

**Affiliations:** 1 National Institute for Sport Medicine, 1113 Budapest, Hungary; 2 Department of Psychology and Health Management, Faculty of Health and Sport Sciences, Széchenyi István University, 9024 Győr, Hungary; boros.szilvia1@sze.hu; 3 Institute of Health Promotion and Sport Sciences, Faculty of Education and Psychology, ELTE Eötvös Loránd University, 1075 Budapest, Hungary; tornoczky.gusztav@ppk.elte.hu; 4 Physical Education and Exercise Centre, Medical School, University of Pécs, 7624 Pécs, Hungary; istvan.karsai@aok.pte.hu; 5 School of Allied Health, La Trobe University, Melbourne 3086, Australia; g.trakman@latrobe.edu.au

**Keywords:** ANSKQ-HU, nutrition knowledge, athletes, education

## Abstract

Nutrition knowledge is essential for optimizing performance, recovery, and overall health in athletes. This study aimed to (1) adapt and validate the Hungarian version of the ANSKQ (Trakman et al., 2017) (ANSKQ-HU) and (2) assess the nutrition knowledge of Hungarian elite and recreational athletes. Following standard translation procedures and expert review, face validity was established. Data were collected from 1.335 athletes, and item difficulty, exploratory factor analysis (EFA), and reliability analyses were performed. A three-factor structure emerged: (1) Fundamentals of nutrition, energy needs, and prohibited substances; (2) Micronutrients and performance-enhancing sports nutrition; and (3) Utilization of macronutrients. While Cronbach’s alpha values were low (α = 0.41–0.62), this seemed acceptable given the dichotomous nature of the questionnaire. Most participants scored poorly (63.3%), with the lowest results in the micronutrients and performance-enhancing nutrition factor. Only 6.9% had formal nutrition education and most frequently respondents received help from coaches, family members, and friends. These findings highlight a significant gap in sports nutrition knowledge among Hungarian athletes and support the need for educative activities organized by sport nutrition professionals. The ANSKQ-HU is a reliable and valid tool for assessing nutrition knowledge in Hungarian athletes and can be a useful questionnaire for their support team (nutritionists, physicians).

## 1. Introduction

Dietary intake plays a significant role in optimizing body composition, enhancing sports performance, and supporting overall health in athletes [1]. Its positive effect exerts not only elite sport but in leisure-time activities as well, as healthy eating and regular exercise play a significant role in health maintenance [2,3,4,5,6]. Nutritional demands vary across various sports and among individuals within the same discipline, influenced by factors such as playing position (in team sports), biological sex, and age [7]. Consequently, personalized strategies developed by qualified sports nutritionists are essential [8,9]. Ideally, these professionals also provide relevant information and education to prevent athletes from relying on unreliable sources and engaging in unsupervised dietary practices [10,11]. Effective nutrition education should start early, ideally when athletes first become involved in sports and including caregivers and coaches to reinforce healthy habits [12,13,14,15]. One of the primary objectives of sports nutrition education is to improve athletes’ dietary choices [16]. Several studies have demonstrated a significant positive relationship between nutrition knowledge and dietary intake [17,18,19]. However, a systematic literature review (SLR) by Heaney et al. (2011) concluded that greater nutritional knowledge does not always result in healthier or more conscious food choices [20]. This finding was supported by a SLR by Spronk et al. (2014), which found only a weak positive correlation (r < 0.5) between nutrition knowledge and food selection [21].

Both Heaney and Spronk suggested that the assessment of the relationship between dietary intake and nutrition knowledge might be negatively affected by inadequate validation of nutrition knowledge questionnaires. In the past decade, several new tools have been developed to evaluate nutrition knowledge [22,23], and the quality of these tools has improved [24]. Among the available tools, the Abridged Nutrition for Sport Knowledge Questionnaire (ANSKQ) is one of the few short-form assessments that evaluates both general and sport nutrition knowledge and is applicable across different sports [25,26,27]. The questionnaire has already been utilized internationally, including among athletes, coaches, and caregivers from Australia [26], the United States [28], Jordan [29], Brazil [17,30,31], the United Kingdom [32], Ireland [18], Greece [33], and Saudi Arabia [34]. In most studies, nutrition knowledge results did not meet acceptable standards, aligning with the original validating questionnaire (47 (SD = 12%)) [26]. Trakman et al. (2019) also noted the existence of Italian, Swedish, Turkish, Chinese, and Malay versions of the questionnaire [35].

To our knowledge, there is currently no reliable and validated Hungarian-language questionnaire available to assess sport nutrition knowledge, and there is also a lack of published research exploring the nutrition knowledge of Hungarian athletes. Since the development of effective educational strategies requires a clear understanding of athletes’ existing level of nutrition knowledge, it is essential to first assess their current understanding using a valid and reliable tool.

Therefore, this present cross-sectional study had two main objectives: (1) to adapt and validate the Hungarian version of the Abridged Nutrition for Sport Knowledge Questionnaire (ANSKQ-HU) for use in athletes, and (2) to evaluate the nutrition knowledge of elite and recreational Hungarian athletes.

## 2. Materials and Methods

### 2.1. Study Design and Ethical Considerations

Ethical approval for the study was granted by the Research Ethics Committee of Eötvös Loránd University’s Faculty of Education and Psychology on 13 November 2023 (Reference No. 2023/479). Following the translation of the ANSKQ, validation and assessment of nutrition knowledge proceeded through data collection by convenience sampling. Participation in the study was voluntary and not associated with any form of compensation. Elite athletes were recruited at the National Institute for Sport Medicine in Budapest, while recreational athletes were recruited from students majoring in sports sciences at the Faculty of Education and Psychology of Eötvös Loránd University, Budapest, and the Faculty of Health and Sports Sciences at Széchenyi István University, Győr. An online circular was distributed to inform participants about the purpose and procedures of the study; informed consent was obtained via a checkbox after scanning a QR code before accessing the questionnaire. Participation was entirely online and self-administered. The same sample was used for the validation and assessment of nutrition knowledge.

### 2.2. English (Abridged) Nutrition for Sport Knowledge Questionnaire and International Adaptations

The original version of the Nutrition for Sport Knowledge Questionnaire (NSKQ) was developed in 2017 and comprised 87 items [27,36]. A shortened version, referred to as the ANSKQ, was released in 2019 to improve completion rates [35]. This version consists of 35 questions, which include general (11 items) and sport-specific (24 items) questions covering various topics such as weight control, macronutrients, micronutrients, dietary supplements, sport nutrition recommendations, and alcohol consumption. Each correct response is assigned one point, resulting in a maximum possible score of 35. Knowledge is categorized as poor if total scores fall below 50%, average between 50–65%, good at 66–75%, and excellent above 75% [35]. The validation process was performed using both Classical Test Theory and Rasch analysis, involving 181 elite Australian athletes engaged in football and netball. Test–retest reliability was established through Spearman’s correlation coefficient (r = 0.7 to 0.8), and construct validity was confirmed using known-group comparisons (*p* < 0.001).

In recent years, adaptations of the NSKQ and ANSKQ have been developed in Arabic (Arabic GSNKQ) [29] and Brazilian Portuguese (NSKQ-BR) [30]. The Arabic version demonstrated excellent internal consistency (Cronbach’s alpha = 0.92), as well as strong test–retest reliability (Pearson’s r = 0.926) and inter-rater agreement (Cohen’s k = 0.89). Two questions were removed from this version due to their religious implications regarding alcohol consumption [29]. The NSKQ-BR was adapted similarly, achieving excellent internal consistency (α = 0.95) and reproducibility, as indicated by an intraclass correlation coefficient (ICC) of 0.85. Notably, the sub-themes of “sports nutrition” and “alcohol” exhibited moderate reproducibility, with ICC values of 0.74 (0.46–0.88) and 0.68 (0.33–0.85), respectively [30].

### 2.3. Development of the Hungarian Abridged Nutrition for Sport Knowledge Questionnaire

Permission to adapt the original questionnaire was acquired on January 24, 2023. In accordance with the questionnaire adaptation methodology outlined by Beaton et al. (2000), two experts independently translated the questionnaire into Hungarian, and discrepancies were resolved through consensus [37]. Following this, an independent expert, who had not seen the original version, conducted a back-translation into English. The resulting English version was then sent to the original author for review, and the Hungarian version was refined based on the feedback received (See Appendice A and Appendix B).

### 2.4. Recruitment and Data Collection

Upon completion of the adaptation process, data collection for the assessment of reliability, validity, and nutrition knowledge was conducted between 1 March 2024, and 31 December 2024. Prior to testing for reliability and validity, an expert group was recruited via a social media platform using convenience sampling. This group was tasked with reviewing and evaluating the applicability of the questionnaire. For the athlete group, sociodemographic data (gender and age) and anthropometric data (self-reported height and body weight, followed by BMI calculation) were also collected. Additionally, participants were asked to report the number of training hours per week, the duration of their involvement in their sport, and the highest competitive level they had attained.

### 2.5. Assessment of Reliability of Hungarian Abridged Nutrition for Sport Knowledge Questionnaire

**Figure 1:**
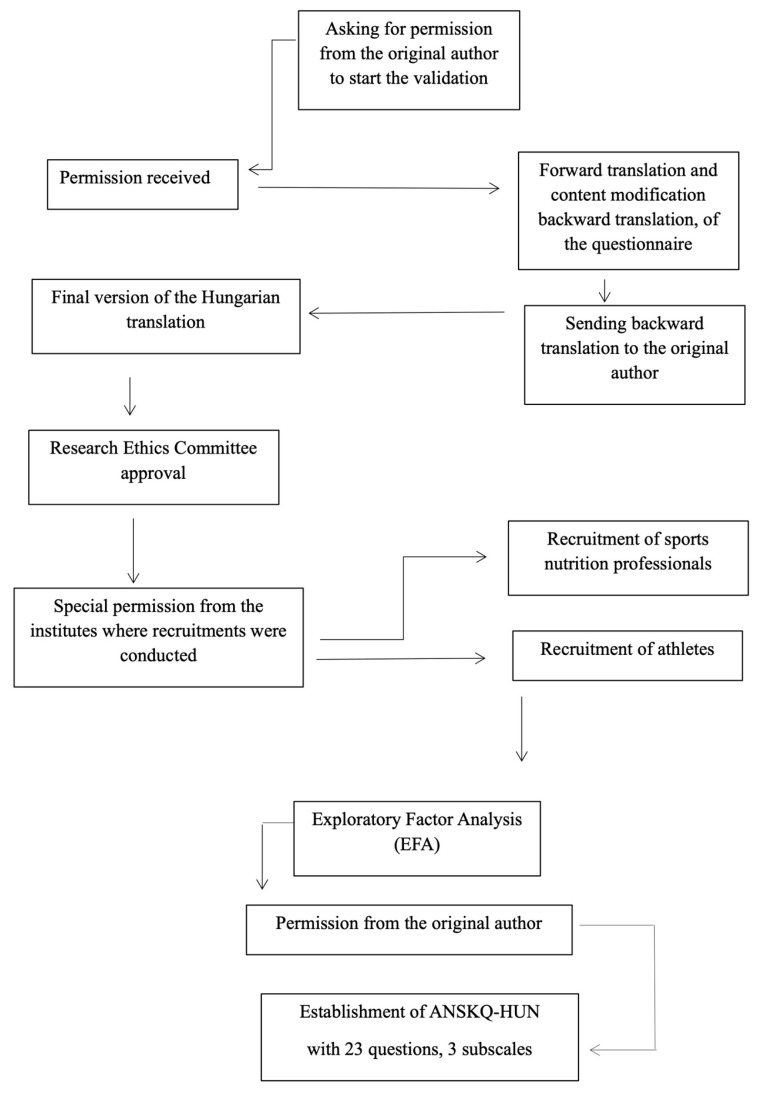
Development and Validation of the Hungarian Abridged Nutrition for Sport Knowledge Questionnaire.

To confirm the reliability and validity of the ANSKQ-HU, face validity, item difficulty, and exploratory factor analysis (EFA) were conducted (refer to statistical analysis). The flow chart detailing the establishment of the ANSKQ-HU is presented in Figure 1.

Figure 1 represents the methodological steps undertaken to adapt and validate the ANSKQ for use in the Hungarian context. Following approval and permission from the original author, the questionnaire was translated into Hungarian through a forward–backward translation procedure, which included minor content modifications to ensure cultural relevance and conceptual equivalence. The back-translated version was reviewed by the original author prior to finalization. Ethical approval was obtained from the Research Ethics Committee, and additional permissions were granted by the institutions where participant recruitment took place. Sports nutrition professionals and both elite and recreational athletes were recruited to participate in the validation process. The final Hungarian version of the ANSKQ, consisting of 23 items across three subscales, was then subjected to Exploratory Factor Analysis (EFA) to examine its construct validity.

## 3. Statistical Analyses

### 3.1. Validation Procedures

Face validity, while not a statistical procedure, is still an important part of the assessment of questionnaires [38]. In this study, face validity was established using the original 35-item version of the ANSKQ-HU, which was approved by Gina Trakman following a post-forward–backward translation process. An expert group of highly qualified representatives from the field evaluated the entire questionnaire using a Likert scale ranging from 1 to 5 (1 = not applicable at all, 5 = highly applicable).

Item difficulty was calculated to examine the validity and psychometric properties of the questionnaire. Item difficulty refers to the proportion of respondents who correctly answered a specific item. This metric is applicable to dichotomously scored items (0 = incorrect response, 1 = correct response), as in the case of the current scale [39]. According to Trakman et al. (2017a) [27], a difficulty index of 0.2 indicates items were answered correctly by less than 20% of the sample and a difficulty index of 0.8 means that items were answered correctly by more than 80% of the sample. Such items should be critically evaluated and considered for removal, with final decisions guided by the relative importance of each item that does not meet the previously mentioned thresholds.

Construct validity refers to the measure of how well a scale accurately measures the trait or theoretical construct it is intended to assess [40]. This construct validity was evaluated through Exploratory Factor Analysis (EFA) utilizing Principal Component Analysis (PCA). The Kaiser–Meyer–Olkin Measure of Sampling Adequacy (KMO values > 0.7 are considered acceptable) and Bartlett’s test of sphericity (significance level *p* < 0.05) were employed to assess the suitability of factor analysis. In accordance with the recommendations of Lorenzo-Seva & Ferrando (2015), in case of scales with dichotomous response formats, the exploratory factor analysis should be based on a polychoric/tetrachoric correlation matrix [41]. Consequently, we followed this methodological approach using JASP 0.95.2 software. An EFA was subsequently conducted via PCA with varimax rotation, utilizing the Kaiser criterion for eigen-values greater than 1 to determine the factor structure.

A reliability analysis was performed to evaluate Cronbach’s Alpha (α) across each subsection identified in the factor analysis and for the scale as a whole [39,42]. The following cut-off points were employed to interpret α > 0.7 as acceptable, >0.6 as questionable, and >0.5 as poor [43]. It is important to note that in nutritional research, low alpha values may occur, for example, in subscales that are well-supported by face validity and are deemed justifiable components of the scale [40]. On the other hand, given the heterogeneous nature of domain-specific knowledge within the ANSKQ-HU, the alpha values should not be considered definitive indicators of validity [44].

### 3.2. Assessment of Participant Characteristics and Nutrition Knowledge

Descriptive statistics, including mean (M), standard deviation (SD), and frequency (%), were used when presenting details regarding the measured variables, specifically demographics and nutrition knowledge scores. The assumption of normal distribution was evaluated through the examination of skewness and kurtosis values. For descriptive statistics, we reported frequencies and mean values with corresponding standard deviations for normally distributed data, while median values and interquartile ranges were utilized for non-normally distributed data. In the statistical analyses, a *p*-value of less than 0.05 was considered statistically significant. All calculations were performed using IBM SPSS 30.0 and JASP 0.95.2.

## 4. Results

### 4.1. Hungarian Version of the ANSKQ: Results of the Final Translated Tool

#### 4.1.1. Face Validity Responses from Sports Nutrition Professionals (Expert Group)

A total of 46 experts consented to participate in the face validity review. Respondents rated the practical applicability of the questionnaire with an average score of 4.18 (SD = 0.92). Two questions related to sport nutrition were identified as problematic, likely due to the use of the unit “cup”, due to grams, decagrams, and kilograms being predominantly used in Hungary. Additionally, regarding the relationship between a diet rich in vitamins and minerals and sports performance, it was suggested that the wording be clarified to emphasize that nutrition alone cannot serve as the sole determinant of athletic performance enhancement. Another comment concerned the question regarding the fat content of margarine, as reduced-fat margarine (20–25%) is also available in Hungary. Nevertheless, at this stage of the development of the Hungarian version, the aforementioned questions were retained in their original form as approved by the original author, given that the comments were given by only 11.5% of the sport nutrition experts. Overall, based on the expert group’s assessments, the content of the ANSKQ-HU is accurately aligned with the intended construct.

#### 4.1.2. Athlete Participant Characteristics

**Figure 2:**
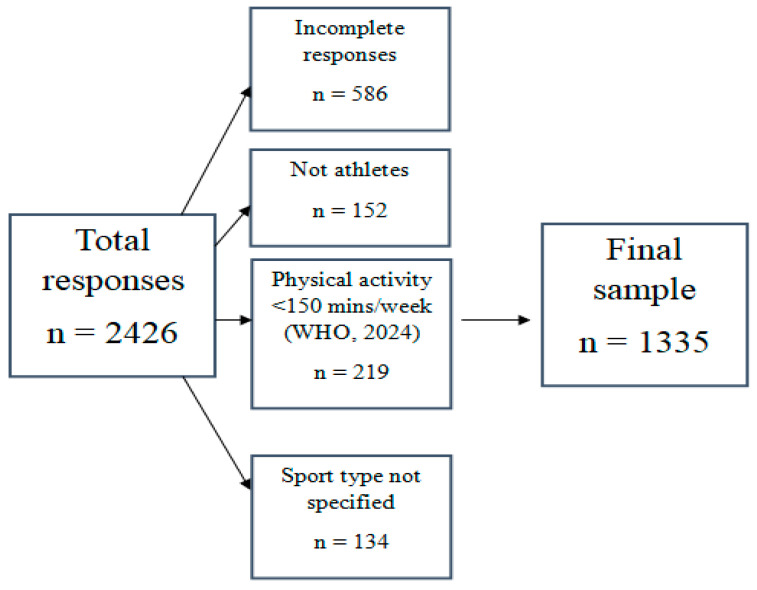
The recruitment of the athlete participants [45].

Further validation and assessment of nutrition knowledge was conducted on athletes. A total of 2426 questionnaire responses were registered. The process for inclusion and exclusion is shown in Figure 2.

The total athlete sample consisted of 1335 participants, encompassing 132 elite athletes and 1203 recreational athletes. Exclusion criteria for respondents included incomplete responses, non-athlete status, engagement in physical activity for less than 150 min per week) [45], and unspecified sport type. The individuals in the final sample formed the basis for descriptive statistics and subsequent analyses.

The mean age of the athlete sample was 22.74 years (SD = 8.11). In terms of gender distribution, 54.3% of the respondents (*n* = 725) identified as male. Regarding the highest competitive level attained, elite athletes predominantly competed at the national and international levels, with proportions of 91.3% and 86.7%, respectively. In contrast, a majority of recreational athletes (41.2%) participated at the national level. Within the entire sample, the largest proportion of respondents (41.21%) reported competing in national-level competitions. Additional demographic variables, including body mass index (BMI) and training hours, are detailed in Table 1.

Participants represented a total of 65 different sports. For data analysis, these sports were categorized into groups based on performance characteristics, as detailed in Table 2.

#### 4.1.3. Item Difficulty

Of the original 35-item scale, the majority of items (n = 29) demonstrated appropriate item difficulty and were therefore included in further statistical analyses. Five items were identified as difficult, while one item was classified as easy (see Table 3). Notably, although the item difficulty values for five original ANSKQ items were below 0.2—designating them as difficult questions—their content provides an opportunity to clarify common misconceptions related to sports nutrition. Consequently, to enhance the practical utility of the questionnaire, the following items were retained: General Nutrition (Gen) 8 and Sport Nutrition (Sport) 12, 13, 20, and 23. This decision aligns with the theoretical framework that the questionnaire evaluates the knowledge of sports nutrition among elite and recreational athletes, thereby identifying potential gaps in their nutritional understanding. In addition to expert group feedback, subsequent exploratory factor analysis also confirmed the significant contribution of these statements/questions to the final version of the ANSKQ-HU, with the exception of one item: General Nutrition 8 (see Table 3). Furthermore, one item (Gen 10) exhibited a difficulty value exceeding 0.8, indicating that it was considered too easy; thus, this item was excluded from further analysis.

#### 4.1.4. Exploratory Factor Analysis (EFA) and Principal Component Analysis (PCA) of the AN-SKQ-HU

The scale employs binary coded responses (0 or 1), and for dichotomous data, it is recommended to utilize a polychoric or tetrachoric correlation matrix based EFA [46]. PCA was performed with varimax rotation to simplify the factor structure by maximizing the variance of the factor loadings. The Kaiser–Meyer–Olkin (KMO) statistic yielded a value of 0.72, and the Bartlett test of sphericity indicated significance (*p* < 0.001), alongside the Chi-squared test (*p* < 0.001), confirming that the assumptions for PCA were satisfied.

Initially, we examined the potential number of distinct factors that could be identified based on eigen-values exceeding 1, considering all items of the ANSKQ scale, with the exception of one item deemed too simplistic (Gen 10). A preliminary number of factors was not specified; however, with a predefined factor loading threshold of 0.4, seven factors were identified. In this model, six items did not meet the requisite conditions, and two factors were defined by only two items each. Subsequently, we evaluated 2 to 5 factor models and analyzed the statistical indicators while taking the theoretical framework into account. Ultimately, the three-factor model emerged as the most suitable representation for the Hungarian version of the scale, a conclusion that was corroborated by the author of the original questionnaire (see Table 4 and Table 5).

The three-factor model comprised 23 items, specifically: Factor 1 included 12 items; Factor 2: 5 items; Factor 3: 6 items (see Table 4). The factors were designated based on contextual relevance as follows: Factor 1: Fundamentals of nutrition, energy requirements of physical activity, and prohibited substances (FEP); Factor 2: Micronutrients and performance-enhancing sports nutrition (MPE); Factor 3: Utilization of macronutrients (UM). Collectively, these three factors account for 35.8% of the total variance.

The original and revised item order is presented to clarify the positioning of each factor component in the updated Hungarian version (see Appendix B). The objective was to prevent the presentation of questions in a sequence that corresponds to the factors; consequently, we preserved their original placements in the questionnaire and excluded only those items not included in the Hungarian version.

#### 4.1.5. Reliability

As Cronbach’s alpha is neither a measure of internal consistency nor a measure of unidimensionality [47], we utilize it as an indicator of reliability. The scale “Fundamentals of nutrition, energy requirements of physical activity, prohibited substances” (FEP) exhibited a Cronbach’s α of 0.62; the scale “Micronutrients, performance-enhancing sports nutrition” (MPE) demonstrated an α of 0.49; the scale “Utilization of macronutrients” (UM) revealed an α of 0.41, and the overall scale yielded an α of 0.62. These values suggest a reliability that ranges from questionable to unacceptable for the scales, as per the criteria established by George & Mallery (2003) [43]. Although reliability testing was performed, it was concluded that this indicator is not suitable for the current questionnaire assessing nutritional knowledge due to specific psychometric considerations. Knowledge-based items typically conform to a formative rather than a reflective measurement model. In formative models, the indicators (i.e., knowledge items) define the construct rather than merely reflect it. Consequently, the overall score is influenced by the presence or absence of specific knowledge components, without presuming that all items tap into a single underlying latent trait [48,49]. For example, an individual may possess knowledge about vitamins but not minerals, or may understand macronutrients but lack awareness of food safety or the regulations of the World Anti-Doping Agency (WADA). These domains of knowledge can be relatively independent; thus, low inter-item correlations do not necessarily signify poor reliability but rather reflect the heterogeneous nature of domain-specific knowledge [46,50]. In light of these considerations, the calculated Cronbach’s alpha values should not be regarded as definitive indicators of the reliability of the questionnaire scales or the overall instrument.

#### 4.1.6. Nutrition Education and Information

Participants were asked whether they had ever studied nutrition, to which 6.9% (*n* = 93) responded affirmatively. A majority of these individuals were female elite athletes (*n* = 49) and their male counterparts (*n* = 31). Regarding sources of information, 70.9% (*n* = 66) of participants reported acquiring nutrition-related knowledge through university courses, 16.1% (*n* = 15) through advanced vocational training, 8.6% (*n* = 8) via online continuing education courses, and four individuals through books.

**Figure 3:**
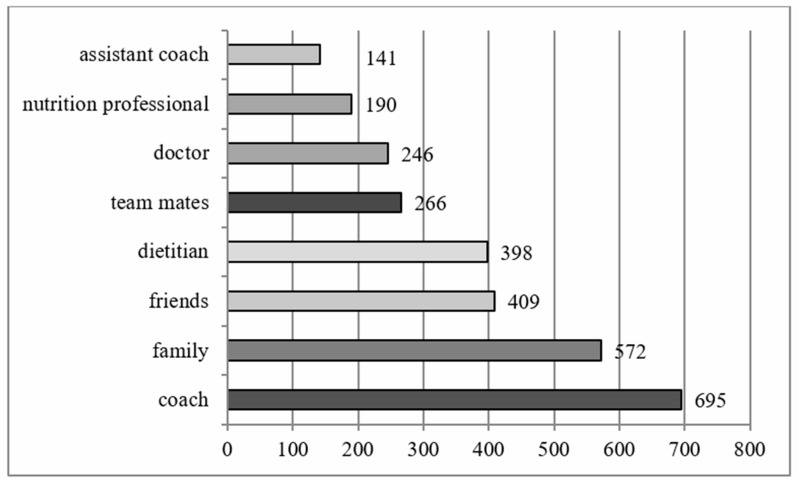
Nutrition-related information sources.

In response to the question of whether they had ever received nutritional advice, only two participants indicated no. Among those who responded affirmatively, 99.8% (*n* = 1333) were able to select multiple sources. The results are summarized in Figure 3.

Respondents most frequently received help from coaches, family members, and friends. Dietitians, teammates, and doctors appeared to be less widespread, while assistant coaches were the least likely to provide nutrition-related information.

#### 4.1.7. Nutrition Knowledge

**Figure 4:**
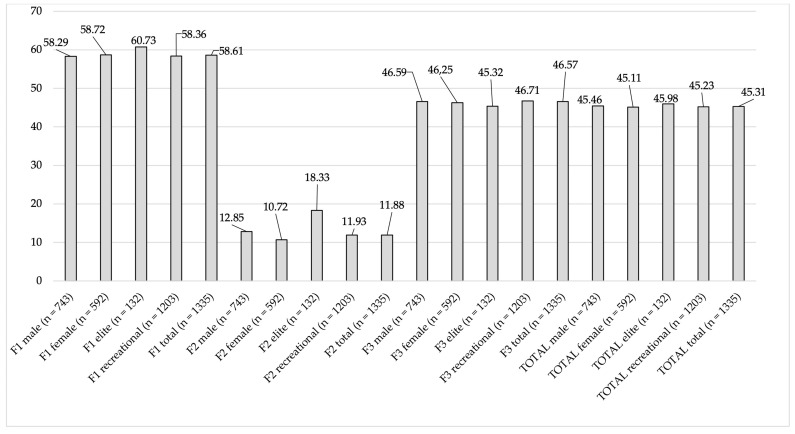
Nutrition knowledge results according to each factor. Note: F1 = Factor 1, F2 = Factor 2, F3 = Factor 3, TOTAL = ANSKQ-HU total scores.

Based on the factor distribution of the original questionnaire, the revised Hungarian version incorporated seven questions pertaining to general nutrition knowledge and sixteen questions related to sports nutrition knowledge. Figure 4 illustrates the results of nutrition knowledge by each factor and total scores according to the revised Hungarian version. The total sample exhibited an average score of 45.31% (SD = 14.71). In terms of categorization, 63.3% of participants were classified as having poor knowledge, 31% as average, 3.9% as good, and 1.8% as excellent. The highest scores were recorded in Factor 1 (Fundamentals of nutrition, energy requirements of physical activity, prohibited substances—FEP), whereas the least favorable results were observed in Factor 2 (Micronutrients, performance-enhancing sports nutrition—MPE). Notably, a statistically significant difference was found in Factor 2 across genders (*p* = 0.03) and competition levels (*p* < 0.01), with women and elite athletes achieving higher scores.

Out of the three new factors, participants met the acceptable level only in the case of F1. Interestingly, the lowest scores were observed in F2, which contains only sport-related questions. In contrast, the other two factors, which also included questions related to general nutritional knowledge, received considerably more favorable responses from the participants.

## 5. Discussion

The objective of this study was to adapt the Abridged Nutrition for Sport Knowledge Questionnaire (ANSKQ) into Hungarian and to evaluate nutrition education, information sources, and nutrition knowledge among a sample of Hungarian athletes. In contrast to the original two-factor structure, exploratory factor analysis (EFA) indicated a modified three-factor model with enhanced psychometric properties.

The expert group found that the translated version of the questionnaire is appropriate for preliminary testing. Following the assessment of face validity, the instrument was subsequently administered to a large sample comprising both recreational and elite athletes. Item difficulty analysis indicated that the majority of the original 35 items exhibited acceptable values; however, some items were identified as difficult but were retained based on expert consensus. One item was deemed excessively easy and was excluded prior to EFA. The resulting 23-item, three-factor model, supported by PCA emerged as clearly distinct, aligning with theoretical expectations and the insights of the author of the original questionnaire. Consequently, a collaboratively developed model is proposed for the final Hungarian version, designated as ANSKQ-HU.

The three factors identified in this model assess distinct domains of nutrition knowledge:

1. Fundamentals of nutrition, energy requirements of physical activity, and prohibited substances (FEP/F1);
2. Micronutrients and performance-enhancing sports nutrition (MPE/F2);
3. Utilization of macronutrients (UM/F3).

Utilizing alternative psychometric assessments, the Hungarian version appears to be a reliable instrument for evaluating nutrition knowledge among athletes. In comparison to previously validated versions of the tool, the Arabic ANSKQ demonstrated strong internal consistency (Cronbach’s alpha = 0.92), test–retest reliability (Pearson’s r = 0.926), and inter-rater agreement (Cohen’s k = 0.89), which were all found to be excellent; additionally, two questions regarding alcohol consumption were removed [29]. Similar findings were reported in the Brazilian adaptation (NSKQ-BR), which exhibited excellent internal consistency (α = 0.95) and reproducibility (intraclass correlation coefficient (ICC) = 0.85) [30]. In our analysis, PCA yielded more favorable results, as out of 35 items, 23 items and 3 factors were confirmed.

According to the ANSKQ-HU scores, the total level of nutrition knowledge was 45.31% (SD = 14.71). A significant proportion of the participants scored poorly (63.3%), which is similar to a Jordanian cross-sectional study, where 88.3% of the sample did not meet the acceptable level [24]. In the same manuscript, 3.2% of the sample scored excellent, which is higher than in our study (1.8%) [29]. The mean total score fell below the minimum 50% cut-off (45.31% (SD = 14.71)), which is similar to an American study where the sample scored an average of 43.7% (SD = 12) [28]. Additionally, an international study focusing on orienteering athletes (*n* = 58) found that only female elite athletes (*n* = 10) reached the acceptable level (52.6% (SD = 13.4)) [51]. Another parallel with this research is that elite athletes scored higher in our case and in the international sample as well [51]. Lower nutrition knowledge scores were reported in the study by Arnaoutis et al. (2024), where handball players (*n* = 39) achieved 38.5% (SD = 10.7) [33]. However, previous studies have also reported higher nutrition knowledge scores. Leen Smith et al. (2025) found that Irish athletes achieved a mean score of 51.6% (SD = 1.37), while Boucherville Pereira et al. (2025) reported that Brazilian athletes reached the acceptable threshold with an average result of 50% [17,18]. These differences may relate to variable access to nutrition education across sporting types and geographic locations, as nutrition education has been shown to improve nutrition knowledge [13].

Interestingly, in our study, athletes achieved generally more favorable results in two factors (F1 and F3) where questions regarding general nutrition knowledge occurred. This may be because athletes are more likely to be exposed to general nutrition education than sports nutrition education through schooling, family, or friends, which were key sources of nutrition education in our cohort.

When investigating the relationship between male and female participants, our Hungarian sample did not show a significant difference between these groups, although males scored slightly higher. However, in the case of FEP/F1, female participants demonstrated better results. In contrast, among Irish athletes, females scored better in general nutrition knowledge but worse in sports nutrition knowledge [18]. Furthermore, Boucherville Pereira et al. (2025) also found that males scored better in sports nutrition knowledge and total scores [17].

Regarding sources of nutrition-related information, athletes reported relying more on coaches, family members, and friends rather than on dietitians or physicians. This finding contrasts with a previous Australian study in which athletes primarily sought nutrition information from dietitians, the internet, and nutritionists [8]. Nevertheless, Elsahoryi et al. (2021) found that athletes tend to use social media (61.8%); one-third of them reported consulting a dietitian (38.1%), and another third sought help from coaches [29]. This present study was limited to athletes, but another noteworthy result from Elsahoryi et al. (2021) concerning nutrition information is that reported coaches relied mostly on social media (49.2%) [29].

## 6. Strengths and Limitations

The key strength of this study is that it is the first Hungarian adaptation of the ANSKQ, performed with an alternative, robust psychometric assessment. Additionally, compared to similar studies, the sample size is relatively large, and the gender distribution is approximately balanced, with athletes from a broad range of disciplines represented. The study fills a significant gap in the literature by providing a new tool for assessing the nutrition knowledge of Hungarian athletes. Similar methods can be applied for additional language and cross-cultural validations.

The face validity testing indicates that the 23-item questionnaire effectively captures the most relevant nutrition knowledge constructs, and the inclusion of difficult items ensures that the tool effectively addresses common misconceptions and fundamental issues through the three subsets of the scale, with previous findings supporting the importance of addressing these knowledge gaps in athletic populations [34].

The validated ANSKQ-HU provides a reliable screening tool for assessing the nutrition knowledge of Hungarian elite and recreational athletes, enabling the identification of specific knowledge gaps. These results can inform the development of targeted nutrition education programs and guide evidence-based interventions for both Hungarian athletes and their support teams, including coaches and caregivers. Furthermore, the tool offers valuable opportunities for Hungarian institutions and sport federations to integrate nutrition education into training curriculum and to evaluate the effectiveness of such programs over time. Finally, the cross-cultural validation of the ANSKQ-HU facilitates future international comparisons with sport-specific research that has also used the ANSKQ/NSKQ, contributing to the global understanding of athletes’ nutrition knowledge.

Despite our efforts to conduct the research as thoroughly as possible, some limitations can be acknowledged. Firstly, completing the questionnaire was self-reported, and although it was clearly stated that the use of aids (e.g., internet, books) was not allowed, we did not independently verify whether participants complied with this instruction. Moreover, criterion validity was not assessed by comparing the ANSKQ-HU with another similar scale, as no validated Hungarian-language instrument is available. Furthermore, we were unable to analyze sport-specific subgroups separately due to the highly heterogeneous sample, which was recruited during an Olympic year that further challenged the elite-group sampling.

## 7. Conclusions

Our tool was unique as it included only 23 items and assessed three distinct factors, as opposed to the more commonly used 35-item, two-factor model. The Hungarian version of the ANSKQ (ANSKQ-HU) demonstrated overall acceptable psychometric properties and a clear three-factor structure, indicating its suitability for assessing sport-related nutrition knowledge in greater detail. Our findings indicate that Hungarian athletes’ nutrition knowledge scores did not differ significantly compared to international data, despite differences in questionnaire structure. Considering all these aspects, the scale is recommended for use by sports professionals, dietitians, and researchers to assess athletes’ nutritional knowledge. Education and prevention play a critical role in promoting optimal nutrition practices, and their significance extends beyond the athletes to encompass their entire support system, particularly coaches and parents. Future interventions should therefore aim to include not only athletes and coaches but also parents and caregivers to maximize their impact. Given these insights, our future research also aims to examine sport-specific differences in nutrition knowledge to identify targeted educational needs. Such an approach may allow for the development of more nuanced, sport-specific strategies to enhance nutritional understanding and ultimately improve athletic performance.

## Figures and Tables

**Table 1:** Anthropometric data, weekly training hours, sports history and competition level data.

Variable	Elite (*n* = 132)		Recreational (*n* = 1203)		Total Sample (*n* = 1335)
Gender	Male (*n* = 57)	Female (*n* = 75)	Male (*n* = 668)	Female (*n* = 535)	
Age	19.89 (SD = 2.63)	20.77 (SD = 3.91)	22.91 (SD = 8.39)	23.13 (SD = 8.45)	22.74 (SD = 8.11)
Height (cm)	184.86 (SD = 8.99)	165.51 (SD = 18.13)	181.61 (SD = 11.19)	168.01 (SD = 6.52)	174.99 (SD = 11.21)
Weight (kgs)	81.45 (SD = 12.35)	59.98 (SD = 8.81)	78.88 (SD = 11.79)	62.16 (SD = 10.11)	70.62 (SD = 10.76)
BMI (kg/m^2^)	24.02 (SD = 4.91)	21.49 (SD = 2.49)	23.93 (SD = 3.27)	21.98 (SD = 3.09)	22.85 (SD = 3.44)
Training hours (per week)	17.41 (SD = 6.60)	14.02 (SD = 6.99)	9.66 (SD = 5.13)	8.61 (SD = 5.02)	12.43 (SD = 5.93)
Years in sport	11.01 (SD = 3.88)	11.06 (SD = 4.51)	10.98 (SD = 6.47)	10.26 (SD = 5.88)	10.82 (SD = 5.19)
HLC (local) (n/%)	-	-	98 (14.7)	95 (17.7)	193 (14.4)
HLC (regional) (n/%)	-	3 (4)	93 (13.9)	48 (8.9)	144 (10.78)
HLC (national) (n/%)	5 (8.7)	7 (9.3)	317 (47.5)	220 (41.2)	549 (41.21)
HLC (international) (n/%)	52 (91.3)	65 (86.7)	160 (23.9)	172 (32.2)	449 (33.6)

**Table 2:** Distribution of sport disciplines based on performance characteristics (*n* = 1335).

Sport Discipline	Elite (*n* = 132)		Recreational (*n* = 1203)		Total Sample (*n* = 1335)
Gender	Male (*n* = 57)	Female (*n* = 75)	Male (*n* = 668)	Female (*n* = 535)	
Aesthetic (n/%)	1 (1.7)	18 (24)	39 (5.9)	133 (24.8)	191 (14.3)
Endurance (n/%)	10 (17.6)	9 (12)	96 (14.3)	117 (21.9)	232 (17.5)
Weight-dependent (n/%)	10 (17.6)	11 (14.7)	72 (10.7)	36 (6.8)	129 (9.6)
Power (n/%)	-	-	2 (0.3)	-	2 (0.14)
Power-endurance (n/%)	16 (28.1)	8 (10.7)	62 (9.3)	25 (4.7)	111 (8.3)
Team (n/%)	17 (29.8)	29 (38.6)	391 (58.6)	222 (41.5)	659 (49.3)
Technical (n/%)	3 (5.2)	-	6 (0.9)	2 (0.3)	11 (0.82)

**Table 3:** The ANSKQ back-translated version with item difficulty values (*n* = 1335).

General Knowledge of Nutrition		ID
1.	The surplus energy consumed from protein may increase the fat content of the body.	0.40
2.	Some body fat is needed in order to recover from diseases.	0.49
3.	In your opinion does Trappista cheese have high or low fat content?	0.78
4.	In your opinion does margarine have high or low fat content?	0.74
5.	In your opinion does honey have high or low fat content?	0.74
6.	The body is able to use protein for the synthesis of muscular protein only to a limited extent.	0.61
7.	Eggs contain all the essential amino acids needed for the body.	0.53
8.	Thiamine (vitamin B1) is necessary for providing the muscles with oxygen.	0.18
9.	Vitamins provide energy (in the form of kilojoule/calorie).	0.49
10.	In your opinion may your bodyweight increase because of alcohol?	0.88
11.	Heavy drinking might be defined as the following:	0.38
Sporting nutrition-based knowledge		
1.	In your opinion does a medium-sized banana contain enough carbohydrates for glucose resynthesis/replacement after an intensive training session?	0.67
2.	In your opinion does one cup of cooked quinoa and one tin of tuna contain enough carbohydrates for the regeneration after an intensive training session?	0.51
3.	In your opinion does 100 grams of chicken breast contain enough protein for muscular growth after an intensive training?	0.36
4.	In your opinion does one cup of cooked beans contain enough protein for muscular growth after an intensive weightlifting training?	0.52
5.	In your opinion does one cup of cooked quinoa contain enough protein for muscular growth after an intensive weightlifting training?	0.53
6.	In order to grow muscles, the most important thing is to consume more protein.	0.23
7.	Which is the most optimal meal to have after-training for those who want to grow muscles (assuming they have already had breakfast and elevenses)?	0.32
8.	If we train at low intensity, our body utilizes mainly fats as a source of energy.	0.48
9.	Vegetarian sportsmen are able to cover their protein needs without food supplements.	0.47
10.	The daily protein need of a sportsman of 100 kg bodyweight, in a well-trained condition, doing exercises of resistance nature, is approximately:	0.49
11.	The optimal calcium intake for sportsmen aged 15–24 years is 500 mg.	0.22
12.	A trained person maintaining a balanced diet may improve their sporting achievement by eating food rich in vitamins and minerals.	0.08
13.	Supplementation of vitamin C for sportsmen is recommended in all cases.	0.12
14.	Sportsmen must drink water to:	0.21
15.	According to experts, sportsmen need:	0.29
16.	Before a competition/race/tournament sportsmen should have such foods which contain much:	0.40
17.	In 60–90-minute-long trainings the consumption of 30–60 g carbohydrates per hour is recommended.	0.34
18.	The consumption of carbohydrates during training helps keep the level of blood glucose stable.	0.58
19.	Which of the following complies the most with the recommendation referring to the snack eaten during an approximately 90-minute-long training of high intensity?	0.45
20.	In your opinion how much protein-consumption is recommended by experts after a training of resistance nature?	0.07
21.	The labels of food supplements sometimes contain such information that is not true.	0.51
22.	All food supplements are tested in order to make sure that they are safe and not contaminated.	0.39
23.	From the following food supplements which is/are said not to have enough proof of improving body composition and improving sport achievement?	0.10
24.	The World Anti-Doping Agency (WADA) bans:	0.70

Note. ID = Item difficulty. Cells with item difficulty less than 0.20 are highlighted with a grey background; cell with more than 0.80 item difficulty is highlighted with light-grey.

**Table 4:** Descriptive statistics of the ANSKQ-HU 3-factor model (*n* = 1335).

Variable	Subscales	Min	Max	M	SD	Sk	Ku
Nutrition for sport knowledge		0	23	10.42	3.38	0.062	0.459
	F1-FEP	0	12	7.03	2.51	−0.285	−0.412
	F2-MPE	0	5	0.59	0.91	1.816	3.514
	F3-UM	0	6	2.79	1.48	0.168	−0.464

Note. M = mean; SD = standard deviation; Sk = skewness; Ku = kurtosis; F1-FEP = Factor 1-Fundamentals of nutrition, energy requirements of physical activity, prohibited substances; F2-MPE = Factor 2-Micronutrients, performance-enhancing sports nutrition; F3-UM = Factor 3-Utilization of macronutrients.

**Table 5:** The principal component analysis of ANSKQ-HU 23 items with orthogonal varimax rotation: three-factor factor loading matrix (*n* = 1335).

Component Loadings					
Item o	Item n	Item H	PC 1	PC 2	PC 3
Gen3	A2	FEP1	0.462	−0.201	0.222
Gen4	A3	FEP2	0.440	−0.288	0.232
Gen5	A4	FEP3	0.517	−0.055	0.240
Gen9	S7	FEP4	0.632	0.258	−0.025
Sport1	S1	FEP5	0.429	−0.166	−0.280
Sport2	S2	FEP6	0.447	0.069	−0.309
Sport4	S4	FEP7	0.489	0.087	−0.069
Sport5	S5	FEP8	0.624	0.215	0.067
Sport10	S7	FEP9	0.404	0.149	0.238
Sport16	S11	FEP10	0.495	0.084	0.086
Sport19	S13	FEP11	0.538	0.007	0.144
Sport24	S16	FEP12	0.579	−0.199	0.190
Sport11	S8	MPE1	0.122	0.586	0.243
Sport12	S9	MPE2	−0.233	0.736	−0.120
Sport13	S10	MPE3	0.067	0.764	−0.103
Sport20	S14	MPE4	0.133	0.659	0.107
Sport23	S15	MPE5	0.075	0.640	0.322
Gen2	A1	UM1	0.078	0.076	0.453
Gen6	A5	UM2	0.282	−0.038	0.455
Gen7	A6	UM3	−0.016	−0.010	0.520
Sport3	S3	UM4	−0.009	0.078	0.485
Sport8	S6	UM5	0.179	−0.015	0.504
Sport17	S12	UM6	0.059	0.124	0.601

Note. Gen = general nutrition knowledge; Sport = sport nutrition knowledge; PC = principal component; Item o = original item abbreviations; Item n = new item abbreviations; Item H = Hungarian scale item abbreviations; FEP = Fundamentals of nutrition, energy requirements of physical activity, prohibited substances; MPE = Micronutrients, performance-enhancing sports nutrition; UM = Utilization of macronutrients. Cells with factor loadings greater than 0.40 are highlighted with a grey background.

## Data Availability

The original contributions presented in this study are included in the article. Further inquiries can be directed to the corresponding author.

## Abbreviations

The following abbreviations are used in this manuscript:

ANSKQ	Abridged Nutrition for Sport Knowledge Questionnaire
ANSKQ-HU	Abridged Nutrition for Sport Knowledge Questionnaire-Hungarian version
SLR	Systematic Literature Review
NSKQ	Nutrition for Sport Knowledge Questionnaire
GSNKQ	General and Sports Nutrition Knowledge Questionnaire
NSKQ-BR	Nutrition for Sport Knowledge Questionnaire-Brazil version
WADA	World Anti-Doping Agency

## Appendix A

Backward translation of ANSKQ

Year of birth:

Gender/Sex:

(a) male
(b) female
(c) other

Country of birth:

Zip code of your present address:

Who do you live with at present?

(a) alone
(b) with parents
(c) with a spouse
(d) with sportsmen
(e) with sportsmen of other sports
(f) other

Have you ever studied human nutrition (eg. university course/training course/online retraining/other diploma)?

(a) yes (please specify)
(b) no

Your highest degree:

Your primary sport discipline:

Your position:

What is the highest level of race/competition which you have ever taken part?

(a) local/of town
(b) of country
(c) national
(d) international

How many hours do you usually train a week (including activities other than your main sport)?

...... hours per week

How many years have you been doing your chosen sport for? (including the years of your primary school, secondary school and university)

……. years

Has any of the following persons ever given you advice on your diet? (You may mark more than one answer.)

(a) trainer
(b) support trainer
(c) dietitian
(d) doctor
(e) friends
(f) family
(g) nutritional science expert
(h) team mates

Rate the 3 most important sources of information which you rely on in relation to your nutrition. Mark them with numbers 1, 2 and 3 in the empty boxes.

(a) scientific periodical
(b) sport trainer/strength-conditioner trainer
(c) leader coach
(d) dietitian
(e) nutritional science expert
(f) doctor
(g) family
(h) friends
(i) searching on the internet (please give the used websites)
(j) media (magazines, radio, television)
(k) social media (Facebook, Twitter)
(l) teammates

Does the sport organization that you are a member of provide access to nutrition-based information or consultation with nutritional science experts/dietitians?

(a) I receive information only in relation to nutrition
(b) I receive information in relation to nutrition as well as possibilities of consulting with nutrition experts/dietitians
(c) neither of the above

In your opinion, do the sports leagues have to ensure the possibility of acquiring knowledge and facts on nutrition and keeping in contact with dietitians/nutritional science experts?

(a) yes, of acquiring knowledge of sports leagues
(b) yes, of acquiring knowledge of nourishment as well as providing the possibility to keep in contact with dietitians/nutritional scientists
(c) no, it isn’t the task of sports leagues

Which of the following types of support would you find useful? Please rate them 1–5.

(a) access to reliable fundamentals and facts relating to nutrients and healthy nourishment
(b) access to reliable, sporting nutrition-based knowledge
(c) partaking in group lectures held by dietitians/nutritional scientists
(d) opportunities of personal/individual consultation with dietitians/nutritional experts
(e) cooking classes/course

Your current bodyweight: ….kg

Your current height: …….cm

Do you take supplements regularly?

(a) yes
(b) no

If yes, what type? ……..

How do you evaluate your knowledge of sport nutrition?

(a) outstanding
(b) above average
(c) below average
(d) insufficient

In your opinion, is it important to eat healthy to improve sports performance?

(a) strongly agree
(b) agree
(c) not agree, neither disagree
(d) disagree

Are you currently studying in higher education?

(a) yes
(b) no

What field of study/university department are you in?

your answer:

GENERAL KNOWLEDGE OF NUTRITION

Gen1. Some body fat is needed in order to recover from diseases.

(a) I agree.
(b) I disagree.
(c) I’m not sure.

Gen2. In your opinion does Trappista cheese have high or low fat content?

(a) high
(b) low
(c) I’m not sure.

Gen3. In your opinion does margarine have high or low fat content?

(a) high
(b) low
(c) I’m not sure.

Gen4. In your opinion does honey have high or low fat content?

(a) high
(b) low
(c) I’m not sure.

Gen5. The body is able to use protein for the synthesis of muscular protein only to a limited extent.

(a) I agree.
(b) I disagree.
(c) I’m not sure.

Gen6. Eggs contain all the essential amino acids needed for the body.

(a) I agree.
(b) I disagree.
(c) I’m not sure.

Gen7. Vitamins provide energy (in the form of kilojoule/calorie).

(a) I agree.
(b) I disagree.
(c) I’m not sure.

SPORTING NUTRITION-BASED KNOWLEDGE

Sport1. In your opinion does a medium-sized banana contain enough carbohydrates for glucose resynthesis/replacement after an intensive training?

(a) enough
(b) not enough
(c) I’m not sure

Sport2. In your opinion does one cup of cooked quinoa and one tin of tuna contain enough carbohydrates for the regeneration after an intensive training?

(a) enough
(b) not enough
(c) I’m not sure

Sport3. In your opinion does 100 g of chicken breast contain enough protein for muscular growth after an intensive training?

(a) enough
(b) not enough
(c) I’m not sure

Sport4. In your opinion does one cup of cooked beans contain enough protein for muscular growth after an intensive weightlifting training?

(a) enough
(b) not enough
(c) I’m not sure

Sport5. In your opinion does one cup of cooked quinoa contain enough protein for muscular growth after an intensive weightlifting training?

(a) enough
(b) not enough
(c) I’m not sure

Sport6. If we train at a low intensity, our bodies utilise mainly fats as a source of energy.

(a) I agree.
(b) I disagree.
(c) I’m not sure.

Sport7. The daily protein need of a sportsman of 100 kg bodyweight, in a well-trained condition, doing exercises of resistance nature, is approximately:

(a) 100 g (1 g/bodyweight kilogram)
(b) 150 g (1,5 g/bodyweight kilogram)
(c) 500 g (5 g/bodyweight kilogram)
(d) should consume as much protein as he/she can
(e) I’m not sure.

Sport8. The optimal calcium intake for sportsmen aged 15–24 years is 500 mg.

(a) I agree.
(b) I disagree.
(c) I’m not sure.

Sport9. A trained person maintaining a balanced diet may improve their sporting achievement by eating food rich in vitamins and minerals.

(a) I agree.
(b) I disagree.
(c) I’m not sure.

Sport10. Supplementation of vitamin C for sportsmen is recommended in all cases.

(a) I agree.
(b) I disagree.
(c) I’m not sure.

Sport11. Before a competition/race/tournament sportsmen should have such foods which contain much:

(a) fluid, fat and carbohydrates
(b) fluid, fiber and carbohydrates
(c) fluid and carbohydrates
(d) I’m not sure

Sport 12. In 60–90-min long trainings the consumption of 30-60 g carbohydrates per hour is recommended.

(a) I agree.
(b) I disagree.
(c) I’m not sure

Sport13. Which of the following complies the most with the recommendation referring to the snack eaten during an approximately 90 min long training of high intensity?

(a) protein shake
(b) mature banana
(c) 2 boiled eggs
(d) one handful of seeds
(e) I’m not sure.

Sport14. In your opinion how much protein-consumption is recommended by experts after a training of resistance nature?

(a) 1.5 g/bodyweight kilogram for most sportsmen
(b) 1 g/bodyweight kilogram for most sportsmen
(c) 0.3 g/bodyweight kilogram for most sportsmen
(d) I’m not sure.

Sport15. From the following food supplements which is/are said not to have enough proof of improving body composition and improving sport achievement?

(a) caffeine
(b) ferulic acid
(c) bicarbonate
(d) leucine
(e) I’m not sure.

Sport16. The World Anti-Doping Agency (WADA) bans:

(a) caffeine
(b) bicarbonate
(c) the use of testosterone
(d) I’m not sure.

Evaluation: Every correct answer equals 1 point and every incorrect answer and ’I’m not sure’ equals 0 point.

Factor 1—Fundamentals of nutrition, energy requirements of physical activity, prohibited substances: Gen2, Gen3, Gen4, Gen7, Sport1, Sport2, Sport4, Sport5, Sport7, Sport11, Sport13, Sport16

Factor 2—Micronutrients, performance-enhancing sports nutrition: Sport8, Sport9, Sport10, Sport14, Sport15

Factor 3—Utilization of macronutrients: Gen1, Gen5, Gen6, Gen3, Gen6, Gen12

Messages in case of online answering

You are a novice in the field of nutrition. You have reached a result of 0–49%. Your knowledge of nutrition needs to be improved. Please move on to the end, where you can check all the correct answers.

You are up to the mark! Your result is 50–65%. Your knowledge about nutrition is average, it needs to be developed in some fields. Please move on to the end, where you can check all the correct answers.

The ace of nutrition! Your result is 66–75%. Your knowledge of nutrition is above the average, ingenious! Please move on to the end, where you can check all the correct answers.

Superstar! Your result is above 75%. Your knowledge about nutrition is outstanding. Please move on to the end, where you can check all the correct answers.

## Appendix B

ANSKQ-HU

Melyik évben született?

Neme:

(a) férfi
(b) nő
(c) egyéb

Melyik országban született?

Mi a jelenlegi postacímének irányítószáma?

Jelenleg kivel él együtt?

(a) egyedül
(b) szülőkkel
(c) párral/házastárssal
(d) sportolókkal
(e) más sportágak sportolóival
(f) egyéb

Végzett valaha humán táplálkozással kapcsolatos tanulmányokat? (pl.: egyetemi kurzus, tanfolyam, online továbbképzés, egyéb diploma)

(a) igen (kérem pontosítsa)
(b) nem

Mi a legmagasabb iskolai végzettsége?

Mi a fő sportága?

Milyen poszton játszik jelenleg?

Mi volt életében a legmagasabb szint, amelyben versenyzett?

(a) helyi/városi
(b) megyei
(c) országos
(d) nemzetközi

Átlagosan hány órát edz egy héten? (beleértve a fő sporton kívüli aktivitásokat is)

……. óra/hét

Hány éve sportol a választott sportágában? (beleértve az általános iskolai, középiskolai és egyetemi éveket is)

…….. éve

Adott-e valaha ezen személyek közül valaki tanácsot az étrendjével kapcsolatban? (Több válasz megadása is lehetséges!)

(a) edző
(b) segédedző
(c) dietetikus
(d) orvos
(e) barátok
(f) család
(g) táplálkozástudományi szakember
(h) csapattársak

Rangsorolja a 3 legfontosabb információforrást, amelyre a táplálkozással kapcsolatban támaszkodik. Jelölje az üres kockákban 1, 2 és 3-as számokkal!

(a) tudományos folyóirat
(b) sportedző/erő-kondícionáló edző
(c) vezető edző
(d) dietetikus
(e) táplálkozástudományi szakember
(f) orvos
(g) család
(h) barátok
(i) internetes keresés (kérjük adja meg a használt webhelyeket)
(j) média (magazinok, rádió, TV)
(k) közösségi média (Facebook, Twitter)
(l) csapattársak

A sportszervezet, melynek tagja, biztosít-e hozzáférést táplálkozással kapcsolatos információkhoz, illetve biztosít-e konzultációt táplálkozástudományi szakemberrel/dietetikussal?

(a) csak táplálkozással kapcsolatos információt kapok
(b) táplálkozással kapcsolatos információt kapok és táplálkozástudományi szakemberrel/dietetikussal is konzultálok
(c) egyik sem

Megítélése szerint a sportszövetségeknek lehetőséget kell biztosítaniuk a táplálkozással kapcsolatos ismeretek elsajátítására és dietetikusokkal/táplálkozástudományi szakemberekkel történő kapcsolattartásra?

(a) igen, a táplálkozással kapcsolatos ismeretek elsajátítására
(b) igen, a táplálkozással kapcsolatos ismeretek elsajátítására és a dietetikusokkal/táplálkozástudományi szakemberekkel történő kapcsolattartásra egyaránt
(c) nem, ez nem a sportszövetségek feladata

Milyen támogatást tartana hasznosnak? Kérjük, rangsorolja 1-től 5-ig az alábbiakat!

hozzáférés tápanyagokkal kapcsolatos megbízható ismeretekhez

hozzáférés sporttáplálkozással kapcsolatos megbízható ismeretekhez

csoportos előadásokon való részvétel, melyet dietetikusok/táplálkozástudományi szakemberek tartanak

egyéni konzultációs lehetőség dietetikusokkal/táplálkozástudományi szakemberekkel

főzőtanfolyam

Jelenlegi testtömege: …..kg

Jelenlegi testmagassága:….. cm

Szed rendszeresen étrend-kiegészítőket? (például: multivitamin, kreatin)

(a) igen (kérem írja le, hogy miket szed jelenleg)
(b) nem

Ha igen, milyet?.....

Hogyan értékeli a sporttáplálkozással kapcsolatos ismereteit?

(a) kiváló
(b) átlag feletti
(c) átlag alatti
(d) hiányos

Véleménye szerint a sportteljesítményhez fontos egészségesen táplálkozni?

(a) nagyon egyetértek
(b) egyetértek
(c) nem értek egyet
(d) nem értek egyet, de nem is cáfolom

Jelenleg felsőoktatásban tanul?

(a) igen
(b) nem

Ha igen, milyen szakon? ……..

ÁLTALÁNOS TÁPLÁLKOZÁSI ISMERETEK

A1. A szervezetnek zsírra van szüksége a betegségek leküzdéséhez.

(a) egyetértek
(b) nem értek egyet
(c) nem vagyok biztos a válaszban

A2. Ön szerint a Trappista sajt magas vagy alacsony zsírtartalmú?

(a) magas
(b) alacsony
(c) nem vagyok biztos a válaszban

A3. Ön szerint a margarin magas vagy alacsony zsírtartalmú?

(a) magas
(b) alacsony
(c) nem vagyok biztos a válaszban

A4. Ön szerint a méz magas vagy alacsony zsírtartalmú?

(a) magas
(b) alacsony
(c) nem vagyok biztos a válaszban

A5. A szervezet korlátozott mértékben képes fehérjét használni az izomfehérje szintézishez.

(a) egyetértek
(b) nem értek egyet
(c) nem vagyok biztos a válaszban

A6. A tojás a szervezet számára szükséges összes esszenciális aminosavat tartalmazza.

(a) egyetértek
(b) nem értek egyet
(c) nem vagyok biztos a válaszban

A7. A vitaminok energiát szolgáltatnak (kilojoule/kalória formájában)

(a) egyetértek
(b) nem értek egyet
(c) nem vagyok biztos a válaszban

SPORTTÁPLÁLKOZÁSSAL KAPCSOLATOS ISMERETEK

S1. Ön szerint egy közepes banán elég szénhidrátot tartalmaz egy intenzív edzés utáni regenerációhoz?

(a) elég
(b) nem elég
(c) nem vagyok biztos a válaszban

S2. Ön szerint egy csésze főtt quinoa és egy konzerv tonhal elég szénhidrátot tartalmaz egy intenzív edzés utáni regenerációhoz?

(a) elég
(b) nem elég
(c) nem vagyok biztos a válaszban

S3. Egy kiadós súlyzós edzést követően ön szerint elég fehérjét tartalmaz 100 g csirkemell az izomnövekedéshez?

(a) elég
(b) nem elég
(c) nem vagyok biztos a válaszban

S4. Egy kiadós súlyzós edzést követően ön szerint 1 csésze főtt bab elég fehérjét tartalmaz az izomnövekedéshez?

(a) elég
(b) nem elég
(c) nem vagyok biztos a válaszban

S5. Egy kiadós súlyzós edzést követően ön szerint 1 csésze főtt quinoa elég fehérjét tartalmaz az izomnövekedéshez?

(a) elég
(b) nem elég
(c) nem vagyok biztos a válaszban

S6. Ha alacsony intenzitáson edzünk, testünk főként zsírokat hasznosít energiaforrásként.

(a) egyetértek
(b) nem értek egyet
(c) nem vagyok biztos a válaszban

S7. Egy 100 kg tömegű, jó edzettségi állapotú, rezisztencia jellegű edzéseket végző sportoló napi fehérje igénye megközelítőleg:

(a) 100 g (1 g/testtömegkilogramm)
(b) 150 g (1.5 g/testtömegkilogramm)
(c) 500 g (5 g/testtömegkilogramm)
(d) fogyasszon annyi fehérjét, amennyit csak tud
(e) nem vagyok biztos a válaszban

S8. Az optimális kalcium bevitel 15–24 éves sportolók részére 500 mg.

(a) egyetértek
(b) nem értek egyet
(c) nem vagyok biztos a válaszban

S9. A kiegyensúlyozott táplálkozást folytató edzett személy vitaminokban, ásványi anyagokban gazdag étkezésekkel javíthatja sportteljesítményét.

(a) egyetértek
(b) nem értek egyet
(c) nem vagyok biztos a válaszban

S10. C-vitamin kiegészítés sportolók részére minden esetben javasolt.

(a) egyetértek
(b) nem értek egyet
(c) nem vagyok biztos a válaszban

S11. Verseny előtt a sportolóknak olyan ételeket javasolt fogyasztani, amiben sok:

(a) folyadék, zsír és szénhidrát
(b) folyadék, rost és szénhidrát
(c) folyadék és szénhidrát van
(d) nem vagyok biztos a válaszban

S12. A 60–90 percig tartó tréningeken, óránként 30–60 g szénhidrát fogyasztása javasolt.

(a) egyetértek
(b) nem értek egyet
(c) nem vagyok biztos a válaszban

S13. Az alábbiak közül melyik felel meg legjobban a körülbelül 90 percig tartó, magas intenzitású edzés során fogyasztott snackre vonatkozó ajánlásoknak?

(a) fehérje turmix
(b) érett banán
(c) 2 főtt tojás
(d) egy marék olajos mag
(e) nem vagyok biztos a válaszban

S14. Ön szerint a szakértők mennyi fehérje fogyasztását javasolják rezisztencia jellegű edzést követően?

(a) 1.5 g/testtömegkilogramm a legtöbb sportoló számára
(b) 1 g/testtömegkilogramm a legtöbb sportoló számára
(c) 0.3 g/testtömeg kilogramm a legtöbb sportoló számára
(d) nem vagyok biztos a válaszban

S15. Melyik étrend-kiegészítőről mondható el, hogy nincs elég bizonyíték arra vonatkozóan, hogy javítja a testösszetételt és fokozza a sportteljesítményt?

(a) koffein
(b) ferulinsav
(c) bikarbonát
(d) leucin
(e) nem vagyok biztos a válaszban

S16. A Nemzetközi Doppingellenes Ügynökség (WADA) tiltja a:

(a) koffein
(b) bikarbonát
(c) karnitin
(d) tesztoszteron használatát
(e) nem vagyok biztos a válaszban

Értékelés: Minden helyes válasz 1 pontot ér, minden helytelen válasz és „nem vagyok biztos a válaszban” 0 pontot ér.

Faktor 1–Általános táplálkozási ismeretek, a fizikai aktivitás energiaigénye, tiltott szerek: A2, A3, A4, A7, S1, S2, S4, S5, S7, S11, S13, S16

Faktor 2–Mikrotápanyagok, teljesítményfokozó sporttáplálkozás: S8, S9, S10, S14, S15

Faktor 3–Makrotápanyagok hasznosulása: A1, A5, A6, S3, S6, S12

Üzenetek online válaszadás esetén

Ön újonc a táplálkozási ismeretek terén. 0–49% közötti eredményt ért el. A táplálkozással kapcsolatos ismeretei fejlesztésre szorulnak. Kérem, haladjon tovább a végéig, ahol minden helyes választ ellenőrizhet.

Megüti a mércét! 50–65% közötti eredményt ért el. A táplálkozással kapcsolatos ismeretei átlagosak, néhány területen fejlesztésre szorulnak. Kérem, haladjon tovább a végéig, ahol minden helyes választ ellenőrizhet.

A táplálkozás ásza! 66–75% közötti eredményt ért el. A táplálkozással kapcsolatos ismeretei átlag felettiek, ügyes! Kérem, haladjon tovább a végéig, ahol minden helyes választ ellenőrizhet.

Szupersztár! 75% feletti eredményt ért el. Kiemelkedőek a táplálkozással kapcsolatos ismeretei. Kérem, haladjon tovább a végéig, ahol minden helyes választ ellenőrizhet.
